# Genic constraint against nonsynonymous variation across the mouse genome

**DOI:** 10.1186/s12864-023-09637-2

**Published:** 2023-09-22

**Authors:** George Powell, Michelle M. Simon, Sara Pulit, Ann-Marie Mallon, Cecilia M. Lindgren

**Affiliations:** 1 Li Ka Shing Centre for Health Information and Discovery, Big Data Institute, University of Oxford, Oxford, UK; 2 Mammalian Genetics Unit, MRC Harwell Institute, Oxfordshire, OX11 0RD UK; 3 Wellcome Centre for Human Genetics, University of Oxford, Oxford, UK; 4 Medical and Population Genetics Program, Broad Institute of MIT and Harvard, Cambridge, MA USA

**Keywords:** Selective constraint, Negative selection, Synonymous and nonsynonymous mutation, Mouse models

## Abstract

**Background:**

Selective constraint, the depletion of variation due to negative selection, provides insights into the functional impact of variants and disease mechanisms. However, its characterization in mice, the most commonly used mammalian model, remains limited. This study aims to quantify mouse gene constraint using a new metric called the nonsynonymous observed expected ratio (NOER) and investigate its relationship with gene function.

**Results:**

NOER was calculated using whole-genome sequencing data from wild mouse populations (*Mus musculus sp* and *Mus spretus*). Positive correlations were observed between mouse gene constraint and the number of associated knockout phenotypes, indicating stronger constraint on pleiotropic genes. Furthermore, mouse gene constraint showed a positive correlation with the number of pathogenic variant sites in their human orthologues, supporting the relevance of mouse models in studying human disease variants.

**Conclusions:**

NOER provides a resource for assessing the fitness consequences of genetic variants in mouse genes and understanding the relationship between gene constraint and function. The study’s findings highlight the importance of pleiotropy in selective constraint and support the utility of mouse models in investigating human disease variants. Further research with larger sample sizes can refine constraint estimates in mice and enable more comprehensive comparisons of constraint between mouse and human orthologues.

**Supplementary Information:**

The online version contains supplementary material available at 10.1186/s12864-023-09637-2.

## Background

Understanding how genetic variation affects organism phenotype and fitness is a fundamental question in biology with implications for biomedical research. Constrained genomic loci (i.e. loci under selective constraint) are depleted of variation due to negative selection and are inferred to be functionally important to organism fitness. Several methods have been used to quantify selective constraint acting on protein-coding genes. The principle of each method is to quantify the degree to which a gene is depleted of amino-acid sequence altering variation relative to the expected variation given neutral evolution [1]. Traditional methods, such as the dN/dS ratio, compare fixed polymorphisms in orthologous genes between species [2]. However, the recent availability of exome sequence data from human populations has enabled researchers to make more powerful, contemporary estimates of selective constraint against different mutation types across human genes. These estimates are valuable as they provide a basis for ranking genes by functional importance (with regard to fitness), interpreting the fitness consequences of mutations, and prioritising candidate mutations that are likely to have a causative role in disease phenotypes [1, 3].

Our understanding of the relationship between gene constraint and gene function is limited by our comprehension of gene constraint in model organisms. Mouse models are crucial for genetic, physiological, and biomedical research, especially in uncovering mammalian gene function and disease mechanisms [4–7]. For instance, mouse models are employed to investigate the in-vivo function of mammalian genes by studying the phenotypic effects of knockouts (i.e. when a gene is rendered inoperative) [8–10]. These knockout experiments have provided insights into the function of constrained human genes, which cannot be directly tested in humans under experimental conditions. However, gene-to-phenotype functional pathways can change over evolutionary time, influencing the extent of selective constraint, and it remains uncertain how they differ between the two species [11–13]. A more comprehensive understanding of mouse gene constraint will elucidate the in-vivo relationships between gene constraint and gene function, and help characterise how constraint has changed between humans and mice in order to prioritise human genes for functional follow-up experiments using mouse models. This could be particularly pertinent for mouse humanization using CRISPR/Cas9 [14], and the clinical development of new drugs [15].

Here, we describe and calculate a new metric for quantifying mouse gene constraint named the nonsynonymous observed expected ratio (NOER), utilising variation from whole-genome sequencing in populations of wild mice (*Mus musculus sp* and *Mus spretus*). We use NOER to assess the relationships between mouse gene constraint and gene function by considering knockout phenotypes from the International Mouse Phenotyping Consortium (IMPC). Furthermore, we highlight the correlation in constraint between mouse and human orthologues, and the enrichment of pathogenic variants in the human orthologues of the most constrained mouse genes. Our metric provides a resource for interpreting the fitness consequences of genetic variants across mouse genes and uncovering the in-vivo relationships between gene constraint and gene function.

## Results

### Quantifying mouse gene constraint against nonsynonymous variation

We quantified selective constraint across 16,609 mouse genes as the nonsynonymous observed expected ratio (NOER), using single nucleotide variants (SNVs) from wild mice populations (*Mus musculus sp.* and *Mus spretus*) [16]. NOER quantifies constraint as the ratio of the observed number of nonsynonymous variants to the expected number given an absence of selection. In brief, the expected number of nonsynonymous variants given no selection in each gene was predicted using a linear model trained on the number of synonymous variants (which are presumed not to have been under selection) as a function of the gene’s mutability and coverage (Supplementary Fig. 3). Mutability and coverage were included as covariates as they can affect the observed number of variants in a gene independent of selection. For example, given an absence of selection, genes that are more mutable are more likely to contain variants. Furthermore, genes with lower coverage have a lower probability of correctly calling variants introducing bias.

We controlled for mutability by calculating methylation status adjusted sequence-specific probabilities of incurring given mutation types (i.e. synonymous or nonsynonymous). Local sequence context (i.e. flanking nucleotides) and the methylation status of CpG dinucleotides affect the substitution probabilities of bases [3]. We calculated probabilities of substitution across noncoding regions of the mouse genome and found the probability of substitution for a given base varies up to 82-fold dependent on its trinucleotide context (i.e. the composition of its flanking bases), and methylated CG dinucleotides are ~ 10-fold enriched for C > T substitutions. We, therefore, calculated the transcript-specific probabilities of synonymous and nonsynonymous substitutions by adjusting for local (trinucleotide) sequence context and the methylation state at CpG sites. Coverage was included as a covariate to account for lower probabilities of substitution detection at loci with low read depth [17]. Genes with lower NOER have relatively fewer nonsynonymous variants than expected given no selection and are considered more constrained. NOER was used to rank genes by their estimated degrees of selective constraint. Genes were binned from 1 to 100 based on their NOER percentile rank, with the most constrained genes in the top percentile and the least constrained in the bottom percentile.

### Gene constraint and knockout phenotype

Gene knockout experiments in mice are used to characterise individual genes by their homozygous complete loss-of-function phenotype and provide a basis for assessing the relationships between gene constraint and gene function. We tested three hypotheses. First, genes associated with severe phenotypes such as embryonic lethality are enriched amongst the most constrained mouse genes. Second, genes with no significant knockout phenotype are enriched amongst the least constrained mouse genes. And third, there is a positive correlation between gene constraint and pleiotropy. We consider homozygous knockout phenotypes from the International Mouse Phenotyping Consortium (IMPC), which characterises mouse knockout phenotypes by measuring various parameters across a range of standardised phenotyping procedures [8].

**Fig. 1:**
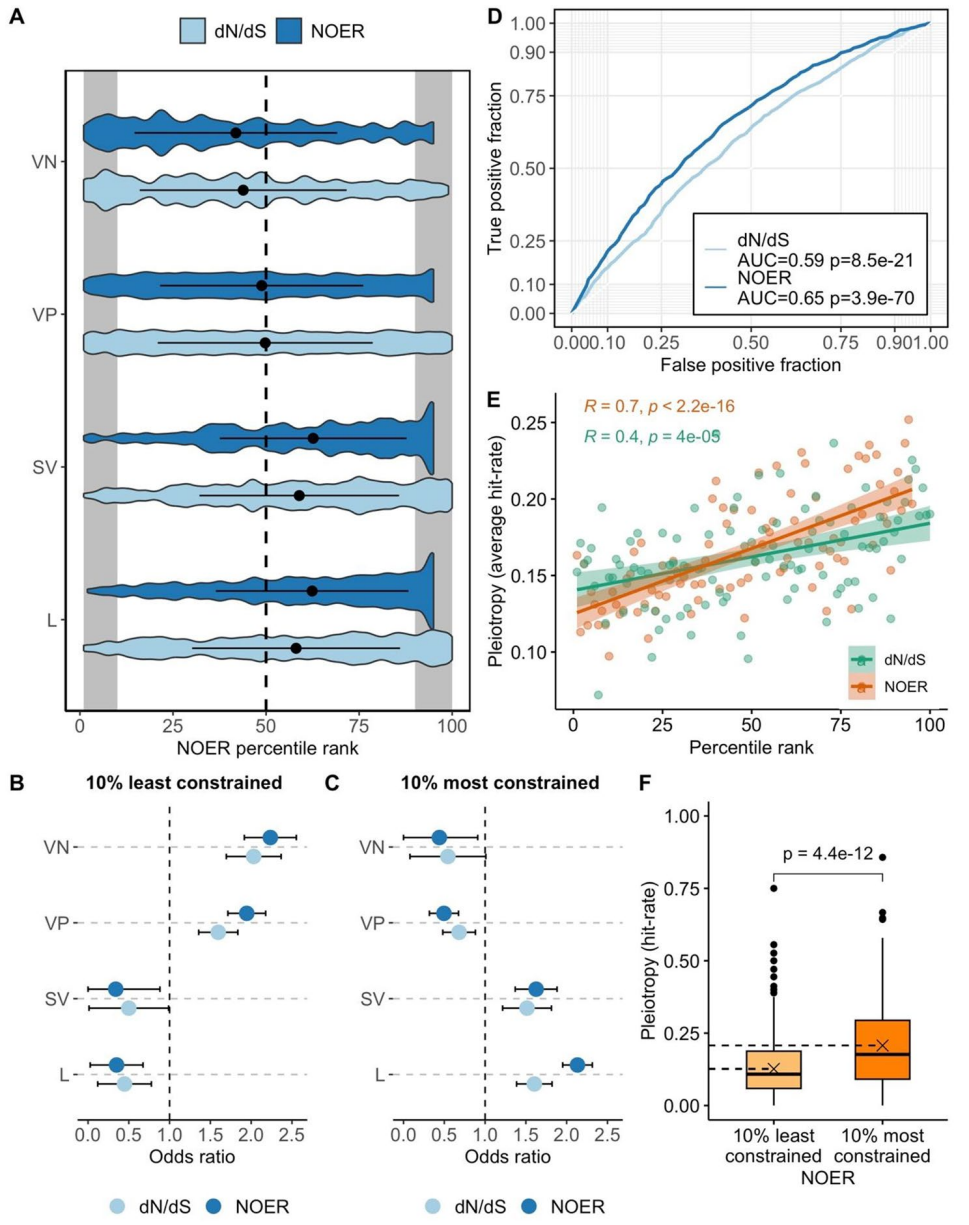
**–** (**A**) Distributions of gene constraint by lethal (L), sub-viable (SV), viable ‘with phenotype’ (VP), and viable ‘no phenotype’ (VN) knockout groupings. Constraint is quantified as the nonsynonymous observed expected ratio (NOER) and dN/dS, with a higher percentile rank indicating a greater degree of constraint. The dashed line indicates the median score for all genes under consideration. The grey areas indicate the 10% most and 10% least constrained genes. (**B** and **C**) Odds ratios and 95% confidence intervals for the 10% most constrained (n = 557), and 10% least constrained (n = 371) genes across each knockout group. An odds ratio greater than one indicates enrichment. The least constrained genes are enriched for genes with no associated phenotype deviation (**B**), and the most constrained genes are enriched for lethal and sub-viable knockout phenotypes (**C**). (**D**) ROC curves highlight the accuracy of NOER and dN/dS to predict knockout lethality or sub-viability. We fit two simple logistic regressions to predict knockout lethality or sub-viability, one with NOER and the other with dN/dS as the explanatory variable. NOER has greater predictive accuracy than dN/dS (0.65 compared with 0.59). (**E**) Scatter plot showing the positive Spearman’s correlation between constraint (NOER and dN/dS) and pleiotropy. Pleiotropy is defined here by hit rate: the number of top-level mouse phenotype ontology terms associated with the knock-out divided by the potential number of top-level terms based on the conducted phenotyping tests. (**F**) The 10% most constrained mouse genes are significantly more pleiotropic than the 10% least constrained mouse genes (p = 4.4e-12). Dashed lines represent the means for each group

We classified 5,252 mouse knockouts conducted by the IMPC into four mutually exclusive categories: lethal (L) if they cause preweaning lethality in all knockouts (n = 1,372); sub-viable (SV) if they cause preweaning lethality in one or more, but not all, knockouts (n = 519); viable phenotype (VP) if they are not classified as lethal or sub-viable, but are annotated with one or more other phenotypes (n = 2,993); and viable ‘no phenotype’ (VN) if they are not annotated with any significant phenotypic deviations from the control group (n = 368). We calculated odds ratios to assess the enrichment of each knockout grouping amongst the 10% most constrained (n = 557) and 10% least constrained (n = 371) IMPC knockouts (Fig. 1B C, Supplementary Fig. 1). Knockouts associated with preweaning lethality or sub-viability are enriched among the most constrained mouse genes (Fig. 1A and B, and 1 C), and knockouts with no annotated phenotype are enriched among the least constrained mouse genes (Fig. 1A and B C). The relative decrease in constraint against nonsynonymous variants in genes with no associated knockout phenotype is likely in part due to these genes having a compensatory network, and/or not playing a critical function in organism fitness. Deviations in phenotype that could result in a fitness cost may, however, have gone undetected as a result of power limitations [18].

We compared NOER with dN/dS to assess its validity. dN/dS is a frequently used measure of the selection effect on a gene, defined as the ratio of nonsynonymous substitutions per nonsynonymous site (dN) to the number of synonymous substitutions per synonymous site (dS) [19]. Genes with a lower dN/dS ratio (i.e. fewer nonsynonymous relative to synonymous substitutions) are considered more constrained, and genes that are neutrally evolving are expected to have a dN/dS ratio of 1. To standardise the comparison, we consider dN/dS scores between *Mus musculus* and *Mus spretus*, as these are the two most distantly diverged species used to calculate NOER. NOER and dN/dS are positively correlated (rho = 0.54, p < 2.2e-16); however, there is limited overlap between the 10% most constrained gene sets defined by each metric (Supplementary Fig. 8). We used simple logistic regression to predict knockouts classified as lethal (L) or sub-viable (SV), and viable ‘no phenotype’ (VN), and assessed predictive accuracy as the area under the receiver operating characteristic curve (AUC). Both NOER (estimate = 0.020 ± 0.001, p = 3.9e-70, df = 5250) and dN/dS (estimate = 0.011 ± 0.001, p = 8.5e-21, df = 3890) are significant predictors of knockout lethality or sub-viability, but predictive accuracy is greater for NOER (AUC = 0.65) compared with dN/dS (AUC = 0.59). Similarly, NOER and dN/dS show the reverse relationship with ‘no phenotype’ knockouts (NOER estimate = -0.016 ± 0.002, p = 9.6e-16, df = 5250; dN/dS estimate = -0.011 ± 0.002, p = 2.4e-7, df = 3890), but predictive accuracy is modestly greater for NOER (AUC = 0.63) compared with dN/dS (AUC = 0.59). The main difference between NOER and dN/dS is their method for estimating the expected number of nonsynonymous variants in each gene given no selection. dN/dS takes the observed synonymous substitution rate to be the expected rate of nonsynonymous substitutions given neutrality, whereas NOER predicts the expected number of nonsynonymous variants using a model. The use of a model to predict the average expected number of nonsynonymous single nucleotide variant (SNV) sites per gene accounts for variance in the rate of neutral evolution between genes [20].

Genes can affect multiple, often seemingly unrelated, phenotypes; a phenomenon known as pleiotropy. Here, we define pleiotropy for each gene as the proportion of phenotypes associated with the IMPC knockout. We calculated pleiotropy for each IMPC knockout as the proportion of phenotyping procedures the knockout was subjected to in which one or more parameters significantly deviated from the control group (i.e. passes a significance threshold of 0.0001). The degree of pleiotropy is positively skewed, with most genes affecting few traits (Supplementary Fig. 2A and 2B). Of the 3,268 knockouts investigated, 368 (11.3%) did not significantly deviate from the control group in any phenotyping parameter, 633 (19.3%) significantly deviated from the control group in one phenotyping parameter, and 2,267 (69.4%) significantly deviated from the control group in more than one phenotyping parameter. Gene constraint is positively correlated with pleiotropy (Spearman’s rho = 0.64, p < 2.2e-16) (Supplementary Fig. 2D; Supplementary Fig. 7A). To test whether this is because genes are affecting closely related phenotypes, we calculated the correlation between gene constraint and the number of associated top-level MP terms (i.e. phenotypes that are more distantly related in the ontology) and found that the relationship persists (Spearman’s rho = 0.7 p < 2.2e-16) (Fig. 1E; Supplementary Fig. 7A). The correlation between gene constraint and pleiotropy may be caused by additive selection effects across multiple phenotypes [21, 22]. For example, the more phenotypes a gene affects, the more likely it is that one or more of the effects caused by functional variation will be deleterious to fitness [23, 24].

### Human pathogenic variant site enrichment

**Fig. 2:**
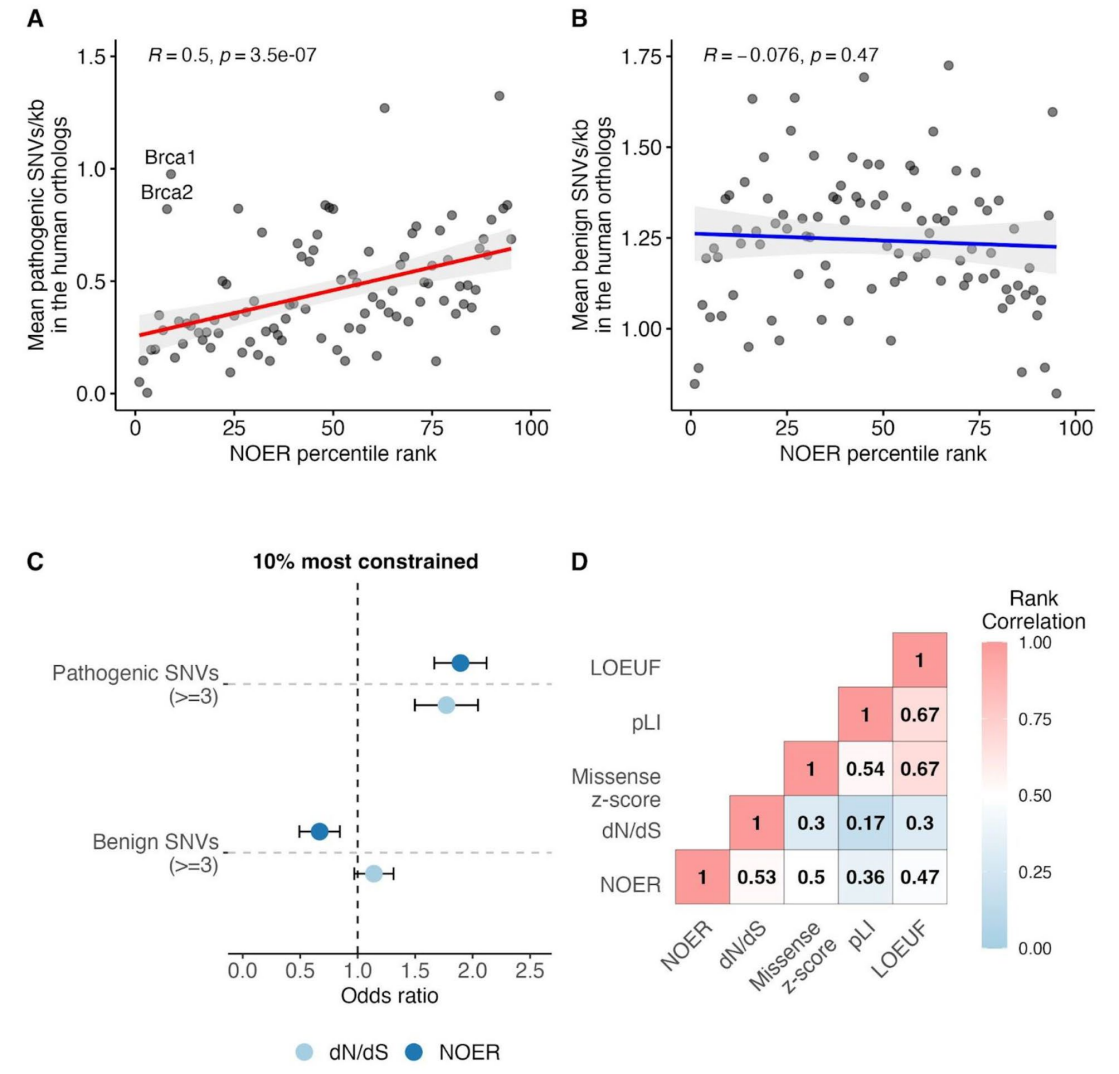
**–** (**A** and **B**) Mouse gene constraint is positively correlated with the number of known pathogenic variants in their human orthologues and negatively correlated with the number of known benign variants. 15,501 pathogenic and 38,423 benign SNV sites were considered from the ClinVar database across 7,016 human genes with a mouse one-to-one orthologue. Constraint is quantified as the NOER, with a greater percentile rank indicating a greater degree of constraint. Rank correlations were calculated between the mean number of pathogenic and benign variants per kb in the human orthologue and the NOER percentile bin. (**C**) Odds ratios and 95% confidence intervals for the enrichment of genes containing 3 or more pathogenic variants in their human orthologue amongst the 10% most constrained mouse genes defined by NOER (n = 1,474) and dN/dS (n = 1,076). The most constrained mouse genes are enriched for human orthologues with 3 or more pathogenic variants and depleted of human orthologues with 3 or more benign variants. (**D**) Spearman’s Rank correlation matrix of constraint scores for human and mouse one-to-one orthologues. Mouse gene constraint is calculated as the nonsynonymous observed expected ratio (NOER), and dN/dS. Human gene constraint scores include the missense *Z*-score, loss-of-function observed/expected upper bound fraction (LOEUF), and the probability of loss-of-function intolerance (pLI). NOER is significantly (Spearman’s rho, p < 0.0001) correlated with metrics of human gene constraint and mouse dN/dS

Mouse models can be used to infer the pathobiology of human disease-associated variants, and their potential for this purpose has increased with the development of CRISPR/Cas9 technologies that enable targeted humanisation of the mouse genome [25]. If selective constraint has been maintained between the human and mouse lineages in part because mutations in constrained genes are more likely to cause disease, it is to be expected that mouse gene constraint will positively correlate with the number of pathogenic variants in their human orthologue. To test this, we considered 16,066 human pathogenic variants from the ClinVar database [26] across 7,016 human genes with a one-to-one mouse orthologue. To account for differences in gene length, we averaged pathogenic variants in each gene per kb. There is a significant positive correlation between mouse gene constraint and the mean number of pathogenic variants per kb in the human orthologue (Spearman’s rho = 0.5, p = 3.53e-7) (Fig. 2A). Furthermore, the 10% most constrained mouse genes are enriched for human orthologues with 3 or more pathogenic variants (OR = 1.90 土0.23) (Fig. 2C). It is possible that this correlation is affected by sampling bias if genes have more recorded pathogenic variants because they have been investigated more thoroughly. To assess this, we also calculated the correlation between mouse gene constraint and the number of benign variants per kb in the human orthologue: if there is sampling bias we would expect to see more benign variants in the genes that have been more thoroughly assessed. However, we observe a non-significant negative correlation between mouse gene constraint and the mean number of benign variants per kb in the human orthologue (Spearman’s rho = -0.08, p = 0.44), and found the 10% most constrained mouse genes are depleted for human orthologues with 3 or more benign variants (OR = 0.67 土0.18) (Fig. 2B and C). This suggests that the result is not an artefact of sampling bias, but rather that the positive correlation between human gene constraint and the number of known pathogenic variants can be in part explained by variation in more constrained genes having an increased likelihood of being pathogenic, and therefore purged by negative selection [1]. Furthermore, the correlation between mouse gene constraint and the number of known pathogenic variants in the human orthologue suggests that negative selection resulting from pathogenicity in these genes may have been maintained between the lineages.

There are, however, some notable outliers to this correlation. For example, *Brca1* and *Brca2* are tolerant of variation, ranking among the 9% ad 8% least constrained mouse genes; however, their human orthologues *BRCA1* and *BRCA2* contain high numbers of pathogenic variants that are causally linked to adult-onset hereditary cancer (Fig. 2A) [27, 28]. Constraint metrics are biased towards the identification of pathogenic variants that cause disease prior to reproduction, and against those that cause disease later in life. This is because selection is likely weaker or non-existent against variants that cause disease after reproduction and therefore do not affect fecundity. These late-affecting pathogenic variants can persist in populations and the ‘healthy’ adult cohorts used to estimate constraint [3].

NOER is positively correlated with metrics of human gene constraint (Fig. 2D), indicating human orthologues of constrained mouse genes are likely to also be constrained. However, there are examples of genes across which the strength of selective constraint substantially differs between the species. *FRAS1-related* extracellular matrix 3 (*FREM3*) is a gene that is highly intolerant of missense variation ranking among the 7% most constrained human genes (measured as the missense z-score). *FREM3* contains no pathogenic variant sites in our analysis but does contain variant sites associated with glycated haemoglobin levels and severe malaria as annotated in the GWAS catalog. By comparison, its murine orthologue *Frem3* is highly tolerant of nonsynonymous variation ranking among the 4% least constrained mouse genes (measured as the NOER). *Frem3* has not yet been knocked out by the IMPC, and currently has no phenotype annotations in the Mouse Genome Informatics database. Calcineurin-like EF-hand protein 2 (*CHP2*) and G protein subunit gamma 13 (*GNG13*) are both highly unconstrained in humans, ranking among the 6% and 9% least constrained human genes (measured as the missense *Z*-score), respectively. *CHP2* and *GNG13* have no disease or phenotype associations annotated by Ensembl and do not contain any pathogenic variant sites in our analysis. By comparison, their murine orthologues *Chp2* and *Gng13* are highly intolerant of nonsynonymous variation ranking amongst the 6% most constrained mouse genes (measured as the NOER). Both *Chp2* and *Gng1* result in sub-viability when knocked out in mice.

It should be noted that there are methodological differences between NOER and human constraint metrics that will result in differences in the information they capture (Supplementary Table 1). For example, human constraint metrics such as the probability of loss-of function intolerance (pLI) and the loss-of-function observed expected ratio (LOEUF) estimate constraint against protein truncating variation. By comparison, NOER predominantly captures constraint against missense variation, as missense variants make up the majority of nonsynonymous variants (99.2%) in the sample used to calculate NOER.

## Discussion

We developed a metric (NOER) to rank mouse genes by their intolerance of nonsynonymous mutation using standing variation between populations of wild mice, and characterised the most constrained mouse genes by their knockout phenotypes. Our research has two important primary findings. First, mouse gene constraint is positively correlated with an increased number of knockout phenotypes, suggesting pleiotropic genes are more likely to be under stronger selective constraint. This may be due to additive selection effects across the multiple phenotypes of pleiotropic genes [21, 22]. For example, if a loss-of-function or gain-of-function variant affects multiple phenotypes, it is likely there will be a net deleterious effect on fitness resulting in selective constraint [23, 24]. This result also has implications for understanding the genetic basis of comorbidity (i.e. the simultaneous presence of two or more diseases in an individual) [29, 30]. Second, mouse gene constraint is positively correlated with an increase in the number of known pathogenic variants in their human orthologues. This correlation suggests that selective constraint can be in part explained by variants in more constrained genes having an increased likelihood of being pathogenic [1] and that this pathogenicity has been maintained between the human and mouse lineages. This could support the use of mouse models for making inferences about the mechanistic functions of human disease variants, which is particularly important given the recent ability to humanise mice with CRISPR-Cas9 technologies [25].

NOER is limited in power and scope by the availability of sequence data from wild mouse populations. The power to detect selective constraint is dependent on the number of variants observed in the sample of sequenced individuals. The more genetic variation in the sample, the greater the power to distinguish between the observed and expected number of variants given neutrality, and therefore estimate constraint. Sequencing of wild mice populations is in its infancy, and the sample sizes are substantially lower compared with sequencing in human populations. For example, NOER is calculated using variation from 67 mice from 8 wild populations whereas contemporary estimates of human genic constraint are based on variation from thousands of individuals [3, 16]. Including genetic variation from *Mus spretus* in the calculation of NOER increases the number of variants in the sample, and therefore the power to estimate constraint. However, *Mus spretus* diverged from the *Mus musculus* lineage approximately two million years ago [31, 32], and this evolutionary distance may introduce error in NOER if constraint has changed between the lineages since their divergence due to changing environments and/or epistasis [33]. Like other constraint metrics, the power for NOER to accurately rank genes by their degree of selective constraint is affected by differences in gene length and mutability. There is more power to estimate constraint in longer, more mutable, genes as they are expected to contain a greater number of neutral variants and therefore they have more scope to be depleted of variation than shorter, less mutable genes. This increases the size of the confidence interval around NOER estimates for shorter genes. Furthermore, as NOER ranks genes by the ratio of observed to expected variants, shorter, less mutable, sequences are more likely to contain no observed nonsynonymous variants by chance and therefore be erroneously classified as maximally constrained (i.e. NOER of 0).

Larger sample sizes of ultra-deep sequencing in wild mice populations would increase the power to calculate constraint metrics and make them more directly comparable to constraint scores calculated from samples of human genetic variation. As larger sample sizes become available, future research could expand on our work to assess selective constraint against rarer, more deleterious mutation types, such as complete loss-of-function mutations, providing insights into haploinsufficiency across the mouse genome. Furthermore, larger sample sizes would enable a more granular assessment of regions within protein-coding sequences that are constrained against missense variation. Gene-wide scores do not capture regional heterogeneity in constraint within protein-coding sequences due to regional differences in the consequences of nonsynonymous variation on protein form and function. Within-gene constrained coding regions have been calculated for human genes, and are enriched for known pathogenic variants [34, 35]. An analogous study in mice would enable the identification of coding regions within mouse genes that are the most intolerant of mutation, and enable between-species comparison of the most functionally important protein domains in orthologous genes. This could inform when the humanisation of mouse genes is appropriate for studying the pathobiology of human variant sites.

## Conclusions

In conclusion, estimates of selective constraint provide insight into the fitness consequences of genetic variation and can be used to prioritise genes by their functional importance. Our research provides a further step toward characterising selective constraint across mouse protein-coding genes. As more data becomes available from larger sample sizes of ultra-deep sequencing in wild mice populations, estimates of selective constraint across the mouse genome will become more powerful and more equivalent for comparison with human constraint scores. Mouse models provide critical insights into mammalian genotype function including for human disease; however, inferences about human biology from mouse models need to be interpreted with an understanding of the evolutionary divergence between the two species. A deeper understanding of how selective pressures have shaped genotype to phenotype pathways in the mouse lineage will continue to shed light on mammalian evolution and provide context for mouse models of human disease.

## Methods

### Raw data and dependencies

Genetic variation (vcf) and coverage (bam) files were downloaded from http://wwwuser.gwdg.de/~evolbio/evolgen/wildmouse/ [16]. *Mus musculus* to *Mus spretus* dN/dS, human to mouse (*Mus musculus*) orthologues, mouse canonical transcript sequences, and genome-wide methylation of CG dinucleotides in mouse embryonic stem cells were downloaded from Ensembl ftp (v94) [36–38]. The soft-masked mouse reference genome (GRCm38), *Mus musculus* to *Mus caroli* pairwise alignment [37], GENCODE exonic annotations [39], the Ensembl Regulatory Build [40], and GERP conserved regions calculated by Ensembl across 111 mammalian species alignments [37] were all downloaded from Ensembl ftp (v101). Measures of intraspecific human constraint, including the missense Z-score [20], loss-of-function observed/expected upper bound fraction (LOEUF) [41], and probability of loss-of-function intolerance (pLI) [42] was downloaded from the supplementary information of their reference papers. Human pathogenic and benign variants were downloaded from ClinVar [26] on 02/03/2021. Knockout phenotypes for 5,252 genes were downloaded from the International Mouse Phenotyping Consortium (IMPC)(release 12.0). All data wrangling, quality control, and analyses were performed using custom scripts written in R (v3.6.2) [43] or bash.

### Defining genes, quality variants, and coding consequences

We considered protein-coding genes with a HUGO Gene Nomenclature Committee name and defined the coding sequence for each gene by their Ensembl canonical transcript (v94) [36]. We excluded transcripts that did not both begin with a start codon and finish with a stop codon, do not have a length that is divisible by three, or contain any character other than A, T, C, or G. Read-depth at every genomic position was calculated for each individual in the sample from the bam files using SAMtools (release 1.11) [44]. Genomic positions were classified as having low coverage if they have a read depth of less than 10X in more than 10% of sequenced mice.

We considered genetic variation from the whole-genome sequencing of 67 mice (*Mus musculus sp*. and *Mus spretus*) from 8 wild populations described in detail by [16]. We considered all autosomal single nucleotide variants (SNV) with ‘PASS’ filter status and classified SNVs as synonymous or nonsynonymous based on their annotated consequences for the amino-acid sequence. The coding consequences of SNVs were determined using the Ensembl Variant Effect Predictor (v94.5) [45]. One consequence was determined per SNV using the “--pick” argument which prioritises annotations by canonical transcript status. Consequences defined as “synonymous_variant”, “stop_retained_variant”, or “start_retained_variant” were classified as synonymous, and consequences defined as “missense_variant”, “stop_gained”, “start_lost”, “stop_lost”, or “protein_altering_variant” were classified as nonsynonymous. After filtering, 501,643 synonymous and 265,343 nonsynonymous (263,303 missense and 2,040 nonsense) SNV sites across 16,609 genes remained for analysis.

### Local sequence context probabilities of substitution

Local sequence context affects probabilities of substitution and has been used to increase the accuracy of models predicting substitution occurrence [3, 20]. We used the trinucleotide context to account for local sequence context, as used by several human metrics [3, 20]. As probabilities of substitution can change over evolutionary time [46], we estimated trinucleotide context probabilities of substitution in the mouse lineage. The trinucleotide context considers one flanking nucleotide on either side of the base of interest. It has 192 parameters as there are 64 possible trinucleotides (4^3^) and the middle base in each trinucleotide has three possible substitutions.

We built a mask for the mouse genome (GRCm38) to filter low coverage regions, repetitive elements, and regions with functional annotation and/or are likely to be under selection. We filtered low coverage loci defined as having less than 10X coverage in more than 10% of individual mice in the sample. Read depth at each genomic position for each mouse in the sample was calculated from the bam coverage files using SAMtools (release 1.11) [44]. We filtered repetitive elements and ambiguous (N) bases by excluding all soft-masked regions classified using RepeatMasker by Ensembl [36]. We also filtered regions more likely to be under selection. These included functional regions annotated as either “promoter”, “promoter flanking”, “enhancer”, “CTCF binding site”, “TF binding site”, or “open chromatin” by the Ensembl Regulatory Build (v101) [40]; all exonic regions annotated by GENCODE [39]; and GERP evolutionarily conserved regions [37]. In total, we masked 75.3% of the mouse genome.

We considered all 41,000,862 single nucleotide variants (SNVs) with “PASS” filter status across unmasked regions of the genome, amounting to approximately 67 SNVs per kb. We inferred the mutational direction of each SNV (i.e. the ancestral and mutant states) using Ensembl’s alignment of the *Mus musculus* (GRCm38) and *Mus caroli* reference genomes. Following previously published protocols [46] the ancestral state for each SNV was assumed to be the reference allele unless the alternate allele was shared with *Mus caroli.*

We calculated the substitution probability for each trinucleotide substitution by dividing the counts of each trinucleotide substitution by the counts of each ancestral trinucleotide across the unmasked ancestral genome (Supplementary Fig. 3A). Each ancestral trinucleotide and trinucleotide substitution has a complementary trinucleotide on the reverse strand. For example, the ancestral trinucleotide TAG on the forward strand must have the complementary trinucleotide CTA on the reverse strand, and a TAG > TTG substitution must result in a complementary CTA > CAA substitution. We, therefore, totalled the counts of each forward and reverse trinucleotide and trinucleotide substitution to calculate substitution probabilities. Trinucleotide substitution probabilities are provided in Supplementary Table 2.

### Methylation states and probabilities of substitution

Unmethylated CG dinucleotides are approximately ten times less likely to undergo C > T substitutions than methylated CGs [47, 48], and accounting for the methylation state has been shown to improve the ability to predict mutation occurrence at CG sites [3]. We, therefore, calculated the substitution rate for methylated and unmethylated CG dinucleotides in the mouse lineage.

Genome-wide methylation of CG dinucleotides in mouse embryonic stem cells was quantified by Stadler et al. (2011) using bisulfite sequencing and were downloaded from Ensembl [36, 38] (v101). Genome-wide methylation data is not available for mouse germline cells. However, similar patterns of methylation have been observed between mouse embryonic stem cells and sperm cells [49], and we, therefore, used mouse embryonic stem cells as a proxy for the germline.

We classified all CG dinucleotides across the unmasked (described above) mouse genome as methylated if they have coverage greater than or equal to 5 and a percentage methylated greater than or equal to 60, and unmethylated if they have coverage greater than or equal to 5 and a percentage methylated less than or equal to 20. We excluded CG dinucleotides with coverage less than 5, or a percentage methylated more than 20 and less than 60. In total, we classified 72.9% of non-masked CG dinucleotides as methylated, 6.9% as unmethylated, and excluded 20.2% from our calculation (Supplementary Fig. 4).

We calculated the probability for cytosine substitutions across three CG dinucleotide groups: all CG dinucleotides, unmethylated CG dinucleotides, and methylated CG dinucleotides. For each unmasked CG dinucleotide, we determined the substitution class by the alternate allele (i.e. C > A, C > G, C > T, G > A, G > C, G > T) (Supplementary Fig. 3B). We calculated the probabilities of substitution for each CG group by dividing the counts of each substitution class in each CG group, by the number of CGs in each CG group. We then combined counts between complementary strands to leave three substitution probabilities: C > A, C > G, and C > T (Supplementary Table 3).

### Transcript-specific probabilities of nonsynonymous and synonymous substitution

There is variation between transcripts in the likelihood that a point substitution will cause either a synonymous or nonsynonymous mutation. This variation is a result of differences in the ratio of synonymous to nonsynonymous mutations that can occur from point substitutions in each codon, and variation in the likelihood of different point substitutions. Each codon has nine possible point substitutions as each of the three bases could be substituted with one of the other three bases (Supplementary Fig. 3C). For example, there is one point substitution to an AAA codon that causes a synonymous mutation and eight that cause nonsynonymous mutations. In comparison, there are three-point substitutions that cause synonymous mutations to CCC codons, and six that cause nonsynonymous mutations. Therefore, a random substitution in a CCC codon is three times more likely to cause a synonymous mutation than a substitution in an AAA codon.

There is further variation in the probability of each point substitution captured by local sequence context, and the methylation state of CG dinucleotides, and accounting for these variables can increase the accuracy of models predicting substitution occurrence [3]. We, therefore, identified unmethylated CG dinucleotides within each transcript. Methylation status for CG dinucleotides was classified from whole-genome bisulfite sequencing in mouse embryonic stem cells, conducted by Stadler et al. (2011) (see above). CG dinucleotides were classified as unmethylated if they have coverage greater than or equal to 5 and a percentage methylated less than or equal to 20. The distribution of CG dinucleotide methylation within transcripts is provided in Supplementary Fig. 4. In total, 18.6% of the CG dinucleotides within the considered transcripts were classified as unmethylated.

We calculated the probability of synonymous mutation for each transcript by: (1) Identifying all possible substitutions that would result in a synonymous mutation across the transcript. (2) Summing the probabilities of substitution for each possible synonymous substitution. Probabilities of substitution for each position in each codon were given by the trinucleotide context; however, for CG dinucleotides in the transcript classified as unmethylated, the probabilities of substitution at the C and G positions are given by the probabilities of substitution calculated for unmethylated CG dinucleotides. This process was repeated to calculate the probability of nonsynonymous mutation for each transcript. A graphical summary of the methods for calculating per gene probabilities of synonymous and nonsynonymous substitution is provided in Supplementary Fig. 3.

### Calculating gene-wide intolerance of nonsynonymous variation

We developed a new metric to quantify gene-wide selective constraint against nonsynonymous variation across the mouse genome, named the nonsynonymous observed expected ratio (NOER). Simply put, NOER captures the ratio of the observed number of nonsynonymous SNVs in a transcript relative to the expected number predicted given no selection. NOER is calculated in a two-stage process (further described in the Supplementary Fig. 3). First, a ‘neutral’ model is trained by fitting a linear regression model to predict the number of synonymous SNVs in each gene as a function of the transcript-specific probability of synonymous mutation and the number of positions classified as ‘low coverage’ in the transcript (see previous subsections). Synonymous SNVs are assumed to be selectively neutral, and so this model estimates the number of SNVs given no selection. The number of synonymous SNVs per transcript ranges from 0 to 435, with a mean of 30.2. The model closely fits the data with an R^2^ of 0.85 and a residual standard error of 11.8 (model coefficients are provided in Supplementary Table 4). Second, the ‘neutral’ model is used to predict the ‘expected’ number of nonsynonymous variants in each gene assuming no selection based on the transcript-specific probabilities of nonsynonymous mutation (Supplementary Fig. 3E). NOER is calculated as the ratio of observed to expected nonsynonymous SNVs. Genes with a score of less than one have fewer nonsynonymous variants than expected given no selection and are considered more constrained. Genes were binned from 1 to 100 based on their NOER percentile rank, with the most constrained genes in the top percentile and the least constrained in the bottom percentile.

The most constrained genes defined by NOER are biased toward either: (1) genes that are short and therefore have a greater probability of containing no nonsynonymous variants by chance and being classified as maximally constrained (i.e. NOER of 0); or (2) longer genes where there is more power to distinguish between the observed and expected number of nonsynonymous variants. We classify the 10% of genes with the lowest NOER scores as highly constrained (n = 1,660). For 306 (18.4%) of these highly constrained genes, the lower bound 95% prediction interval of the expected number of nonsynonymous variants is less than or equal to 0 (i.e. they have a > = 2.5% chance of containing no nonsynonymous variants based on the neutral model).

A gene must be predicted to contain a minimum of 35 nonsynonymous variants by the neutral model to appear amongst the 10% most constrained genes if they are observed to contain one nonsynonymous variant. Of the 16,609 genes assessed, 4,133 (24.9%) have an expected number of nonsynonymous variants below this threshold. Distributions of gene length by NOER percentile are provided in Supplementary Fig. 5. Distributions of synonymous and nonsynonymous counts by transcript are provided in Supplementary Fig. 6.

### Assessing mouse gene constraint and knockout phenotype

We investigated the relationship between gene constraint measured as NOER and gene function, by considering knockout phenotypes for 5,252 genes from the International Mouse Phenotyping Consortium (IMPC)(release 12.0). The IMPC is systematically knocking out all protein-coding genes across the mouse genome. Each knockout in the IMPC is subject to a standardised set of phenotyping procedures at set time points [8]. Each procedure tests multiple parameters, and these parameters are tested for statistical deviation from a control group of non-knockout mice. Parameters that statistically deviate from the control are annotated with Mouse Phenotype Ontology (MP) terms [9].

We classified 5,252 knockouts with a NOER into one of four categories. Knockouts were classified as lethal (L) if they are annotated as causing preweaning lethality in all knockouts (n = 1,372); sub-viable (SV) if they are annotated as causing preweaning lethality in one or more, but not all, knockouts (n = 519); viable phenotype (VP) if they are not classified as L or SV, but are annotated with one or more other MP term (n = 2,993); and viable ‘no phenotype’ (VN) if they were subjected to one or more phenotyping procedure but are not annotated with any MP terms (n = 368) (i.e. knockouts that did not pass a significance threshold of 0.0001 in one of the associated phenotyping parameters). We calculated odds ratios to analyse gene set enrichment for each grouping in the 10% most constrained IMPC knockouts (i.e. nonsynonymous *Z*-score percentile > = 91), and the 10% least constrained genes (i.e. nonsynonymous *Z*-score percentile < = 10). For example, to assess the enrichment of L knockouts among the most constrained genes we used the formula:

$${\rm{OR} = (\rm{A}/\rm{B})/(\rm{C}/\rm{D})}$$

Where A is the number of knockouts classified as L with a NOER percentile > = 91; B is the number of genes not classified as L with a NOER percentile > = 91; C is the number of genes classified as L with a NOER percentile < 91; D is the number of genes not classified as L with a NOER percentile < 91. 95% confidence intervals were calculated as:

$${1.96*\rm{s}\rm{q}\rm{r}\rm{t}((1/\rm{A}) + (1/\rm{B}) + (1/\rm{C}) + (1/\rm{D})).}$$

We fit simple logistic regression models to predict knockouts classified as L or SV, and knockouts classified as VN, as a function of gene constraint (measured as NOER and dN/dS). We assessed model fit and predictive accuracy as the area under the receiver operating characteristic (AUC).

We defined pleiotropy as the proportion of phenotypes associated with the IMPC knockout. Two measures of pleiotropy were calculated for each IMPC knockout. First, the proportion of parameters tested that significantly deviate from the control group (i.e. passes a significance threshold of 0.0001). Second, the number of top-level mouse phenotype ontology terms associated with the knock-out, divided by the potential number of top-level terms based on the conducted phenotyping tests. We filtered knockouts that cause lethality, sub-viability, or were subject to less than five phenotyping tests. We calculated two Spearman’s Rank correlations (1) between the median proportion of phenotyping procedures with one or more significant parameters per mouse knockout and the NOER percentile bin, and (2) between the median proportion of unique top-level mouse phenotype ontology terms per mouse knockout and the NOER percentile bin. The MP ontology is a directed acyclic graph, and it is possible for one MP term to have multiple top-level terms. We, therefore, counted only one top-level MP term per MP term.

### Defining orthologues and correlation with other measures of constraint

Human-mouse orthologues were defined by Ensembl (v94) [36]. We tested whether mouse gene constraint measured as NOER was correlated (Spearman’s rank) with *Mus musculus* to *Mus spretus* dN/dS and previously published measures of intraspecific human constraint, including the missense *Z*-score [20], LOEUF [41], and pLI [42].

### Assessing pathogenic variant enrichment in the human orthologues

Human pathogenic and benign variants were downloaded from ClinVar [26] on 02/03/2021. We classified SNV sites with clinical significance labelled as either “Pathogenic” or “Likely pathogenic”, as pathogenic, and those with “Benign” or “Likely benign” as benign. All benign and pathogenic SNVs considered have a review status labelled as either “criteria provided, multiple submitters, no conflicts”, “criteria provided, single submitter”, or “reviewed by expert panel”. The Ensembl canonical transcripts and coding consequences for these SNVs were determined using the Ensembl Variant Effect Predictor (v94.5) [45]. We considered SNVs with consequences defined as either “synonymous_variant”, “stop_retained_variant”, “start_retained_variant” “missense_variant”, “stop_gained”, “start_lost”, “stop_lost”, or “protein_altering_variant”. We considered human genes with one or more pathogenic or benign variant sites, and with a one-to-one mouse orthologue for which NOER is calculated. This left 16,066 pathogenic variant sites and 38,423 benign variant sites for analysis across 7,016 human genes. To account for differences in gene length, we averaged pathogenic variants in each human gene per kb. We calculated Spearman’s rank correlations between the mean number of pathogenic variants per kb and the NOER percentile bin and the mean number of benign variants per kb and the NOER percentile bin.

### Electronic supplementary material

Below is the link to the electronic supplementary material.

Supplementary Material 1

Supplementary Material 2

## Data Availability

All data analysed during this study are publicly available. Genetic variation (vcf) and coverage (bam) files are available from http://wwwuser.gwdg.de/~evolbio/evolgen/wildmouse/ [16]. *Mus musculus* to *Mus spretus* dN/dS, human to mouse (*Mus musculus*) orthologues, mouse canonical transcript sequences, and genome-wide methylation of CG dinucleotides in mouse embryonic stem cells are available from Ensembl ftp (v94) (https://ftp.ensembl.org/pub/release-94/) [36–38]. The soft-masked mouse reference genome (GRCm38), *Mus musculus* to *Mus caroli* pairwise alignment [37], GENCODE exonic annotations [39], the Ensembl Regulatory Build [40], and GERP conserved regions calculated by Ensembl across 111 mammalian species alignments [37] are available from Ensembl ftp (v101) (https://ftp.ensembl.org/pub/release-101/). Human pathogenic and benign variants are available from ClinVar [26] (https://ftp.ncbi.nlm.nih.gov/pub/clinvar/). Knockout phenotypes for 5,252 genes are available from the International Mouse Phenotyping Consortium (IMPC) (release 12.0) (http://ftp.ebi.ac.uk/pub/databases/impc/all-data-releases/release-12.0/). A combined dataset of the variables used in the final analyses is provided in the Supplementary Table 5.
