# Supplementary material for: Genic constraint against nonsynonymous variation across the mouse genome
Source: BMC Genomics. 2023 Sep 22;24:562. doi: 10.1186/s12864-023-09637-2 (PMC10514939; doi:10.1186/s12864-023-09637-2)
Supplement: Supplementary file 1 — Supplementary Material 1 [file 12864_2023_9637_MOESM1_ESM.pdf]

Supplementary Material

Table of Contents

Supplementary Figure 1 2
Supplementary Figure 2 3
Supplementary Figure 3 6
Supplementary Figure 4 7
Supplementary Figure 5 8
Supplementary Figure 6 9
Supplementary Figure 7 10
Supplementary Figure 8 11
Supplementary Table 1........ 12
Supplementary Table 2........ 14
Supplementary Table 3........ 16
Supplementary Table 4........ 16

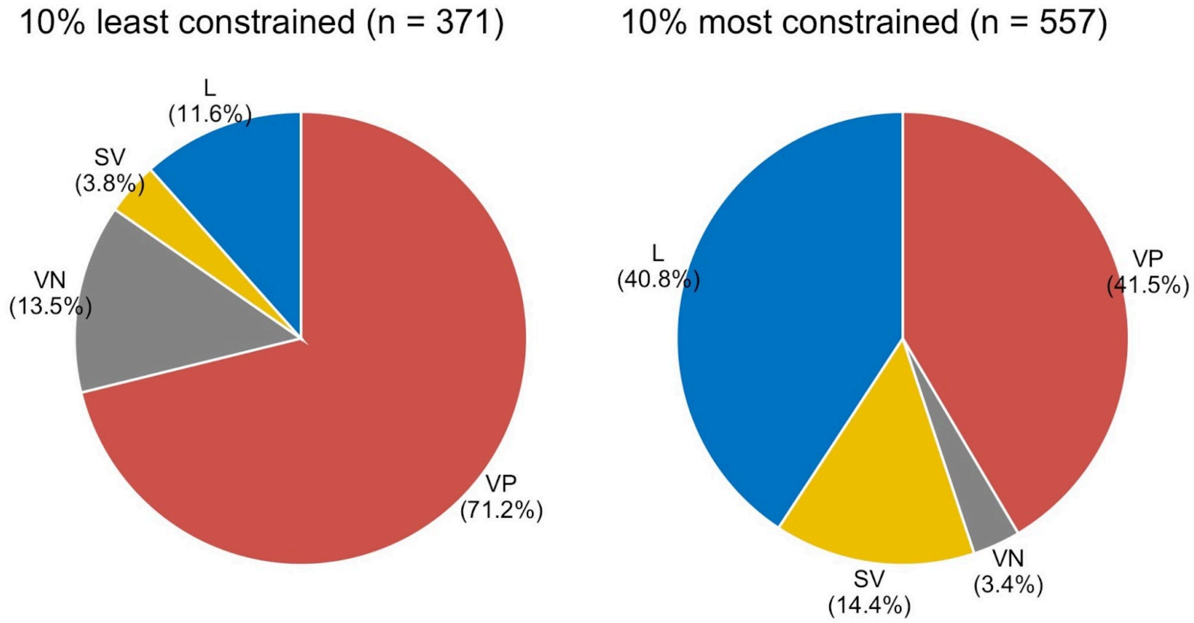

Supplementary Figure 1 - Compositions of the 10% most and 10% least constrained gene sets (defined by NOER) by knockout groupings: lethal (L), sub-viable (SV), viable ‘with phenotype’ (VP), and viable ‘no phenotype’ (VN).

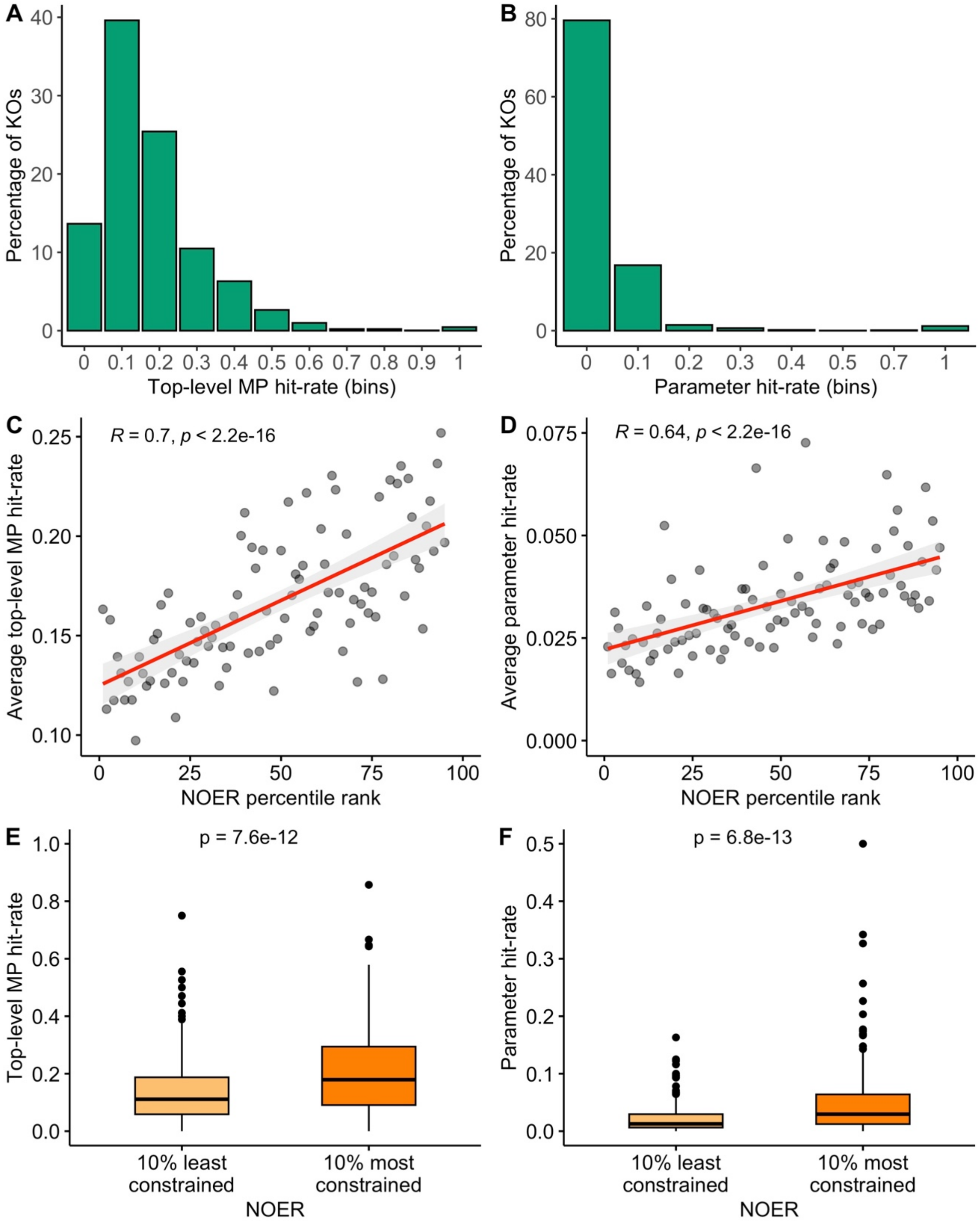

Supplementary Figure 2 – Percentage of knockouts (KOs) by hit-rate bins defined by top-level mouse phenotype (MP) and parameter (A and B). Spearman's rank correlation between NOER percentile and average hit-rate defined by top-level MP and parameter (C and D). Differences in the pleiotropy hit-rate defined by top-level MP and parameter for the 10% least and 10% most constrained genes (E and F).

A. Calculate trinucleotide probabilities of substitution across intergenic (non-filtered) regions of the mouse genome.

Example:

Ancestral: A A A A A A T
Mutant: A C A A A T T

Ancestral trinucleotide	Mutant trinucleotide	N ancestral	N substitution
AAA	ACA	4	1
AAA	AGA	4	0
AAA	ATA	4	0
AAT	ACT	1	0
AAT	AGT	1	0
AAT	ATT	1	1

Trinucleotide probabilities of substitution $P(XY_1Z \rightarrow XY_2Z)$ are calculated as:

$P(XY_1Z \rightarrow XY_2Z) = A/B$

Where B is the number of trinucleotide XY_1Z in the ancestral sequence, and A is the number of $XY_1Z \rightarrow XY_2Z$ changes.

B. Calculate probabilities of substitution for unmethylated CG dinucleotides across intergenic (non-filtered) regions of the mouse genome.

Example:

Methylation: + + + + - -
Ancestral: C G C G A C G
Mutant: T G C G A C G

Ancestral	Mutant	Methylation state	N ancestral	N substitution
CG	AG	+	2	0
CG	GG	+	2	0
CG	TG	+	2	1
CG	AG	-	1	0
CG	GG	-	1	0

CG	TG	-	1	0

Unmethylated CG probabilities of substitution $P(Y_1Z \rightarrow Y_2Z)$ are calculated as:

$P(Y_1Z \rightarrow Y_2Z) = A/B$

Where B is the number of unmethylated CGs (Y_1Z) in the ancestral sequence, and A is the number of $Y_1Z \rightarrow Y_2Z$ changes.

B. Use probabilities of substitution to calculate per gene probabilities of synonymous and nonsynonymous substitution.

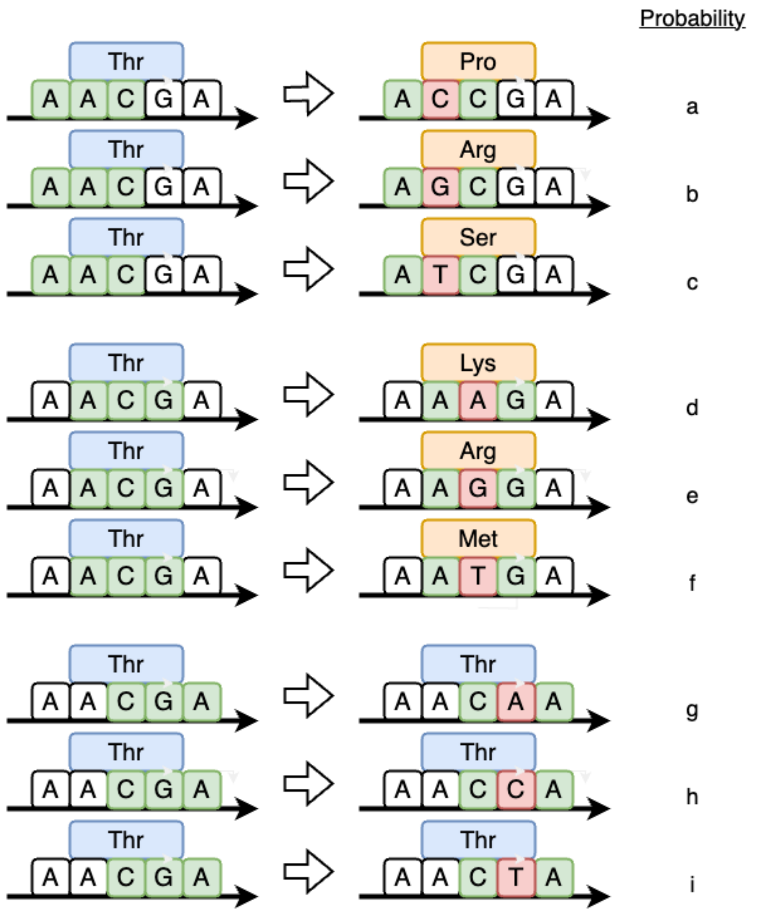

For each gene, the probability of synonymous and nonsynonymous substitution is calculated by summing the probabilities of all possible substitutions at each position that would result in the given mutation type. For example, the probability of synonymous substitution for the Thymine in the above sequence is:

P(synonymous) = g + h + i

The probability of nonsynonymous substitution for the Thymine is:

P(nonsynonymous) = a + b + c + d + e + f

Note, probabilities *a* to *i* are based on the trinucleotide context. If the CG in the ACG codon is unmethylated, the probabilities of substitution at the C and G positions would be given by

the probabilities of substitution calculated for unmethylated CG dinucleotides as opposed to the trinucleotide context.

C. Train a 'neutral' model by regressing the number of synonymous SNVs in each gene against 1) the gene-specific probability of synonymous mutation and 2) the number of positions classified as 'low coverage' in the gene.

$$y = \beta_0 + \beta_1 x_1 + \beta_2 x_2 + \varepsilon$$

Where y is the number of synonymous SNVs in each gene; x_1 is the transcript-specific probability of synonymous mutation; and x_2 is the number of positions classified as 'low coverage' in the gene. β_0, β_1, and β_2 are the regression coefficients, representing the intercept and slopes of the regression line for each predictor variable. ε is the error term, representing the random variability or noise in the model.

D. Use the 'neutral' model to predict the 'expected' number of nonsynonymous variants in each gene assuming no selection by switching the transcript-specific probability of synonymous mutation with the transcript-specific probability of nonsynonymous mutation.

$$\hat{y} = \beta_0 + \beta_1 x_1 + \beta_2 x_2$$

Where $\hat{y}$ is the predicted number of nonsynonymous SNVs in each gene; x_1 is the transcript-specific probability of nonsynonymous mutation; and x_2 is the number of positions classified as 'low coverage' in the gene. β_0, β_1, and β_2 are the regression coefficients, representing the intercept and slopes of the regression line for each predictor variable from the 'neutral' model.

E. Calculate NOER as:

NOER = Obs / Exp

Where Obs is the observed number of nonsynonymous variants in the gene, and Exp is the number of nonsynonymous variants predicted for the gene with the neutral model.

Supplementary Figure 3 – Graphical outline of the methods for calculating per gene probabilities of synonymous and nonsynonymous substitution and NOER.

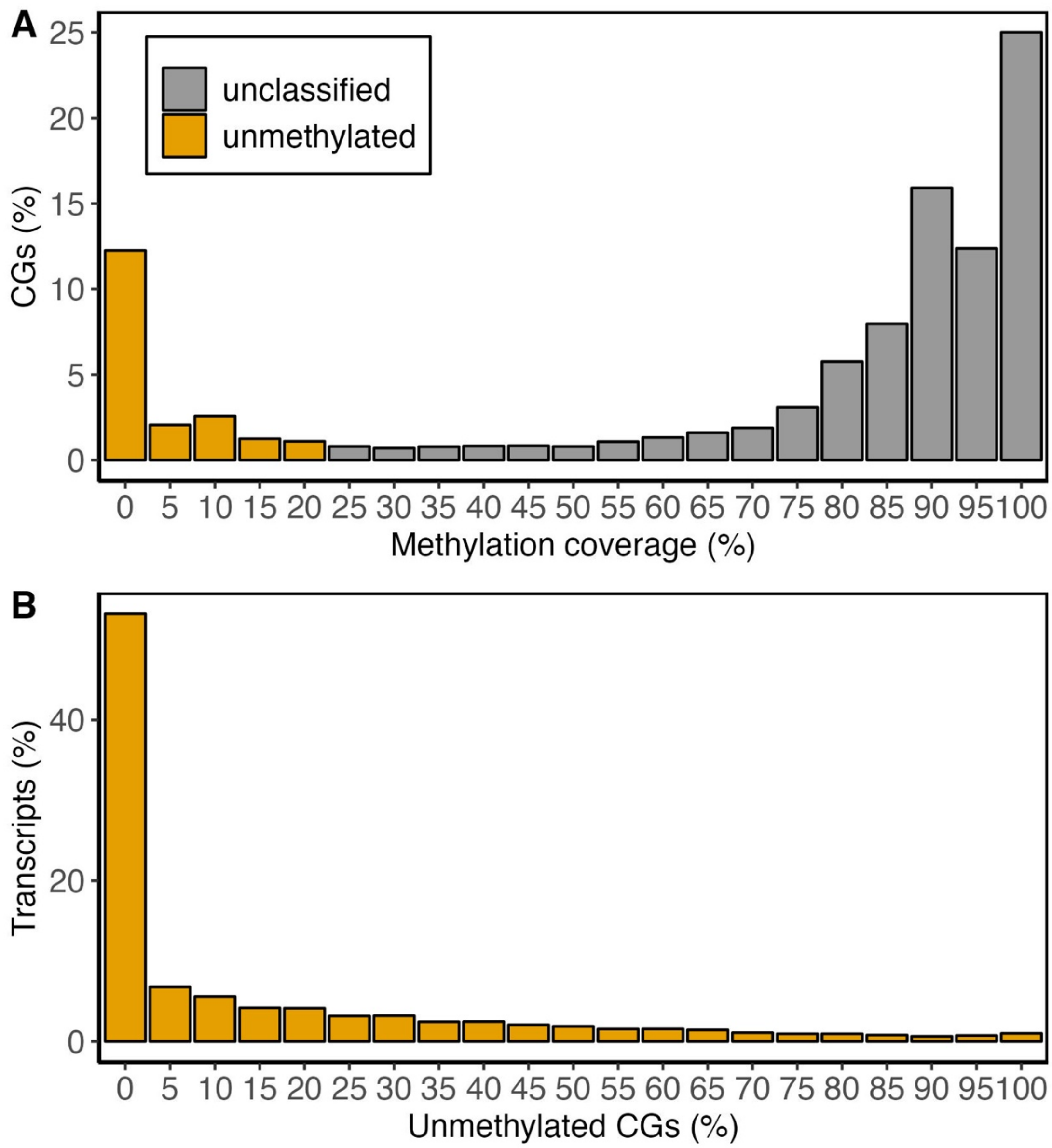

Supplementary Figure 4 – A) Bisulphate methylation coverage across the mouse genome. CG dinucleotides were classified as unmethylated if they have a coverage greater than or equal to 5 and a percentage methylated less than or equal to 20. B) Distribution of CG methylation status across mouse transcripts.

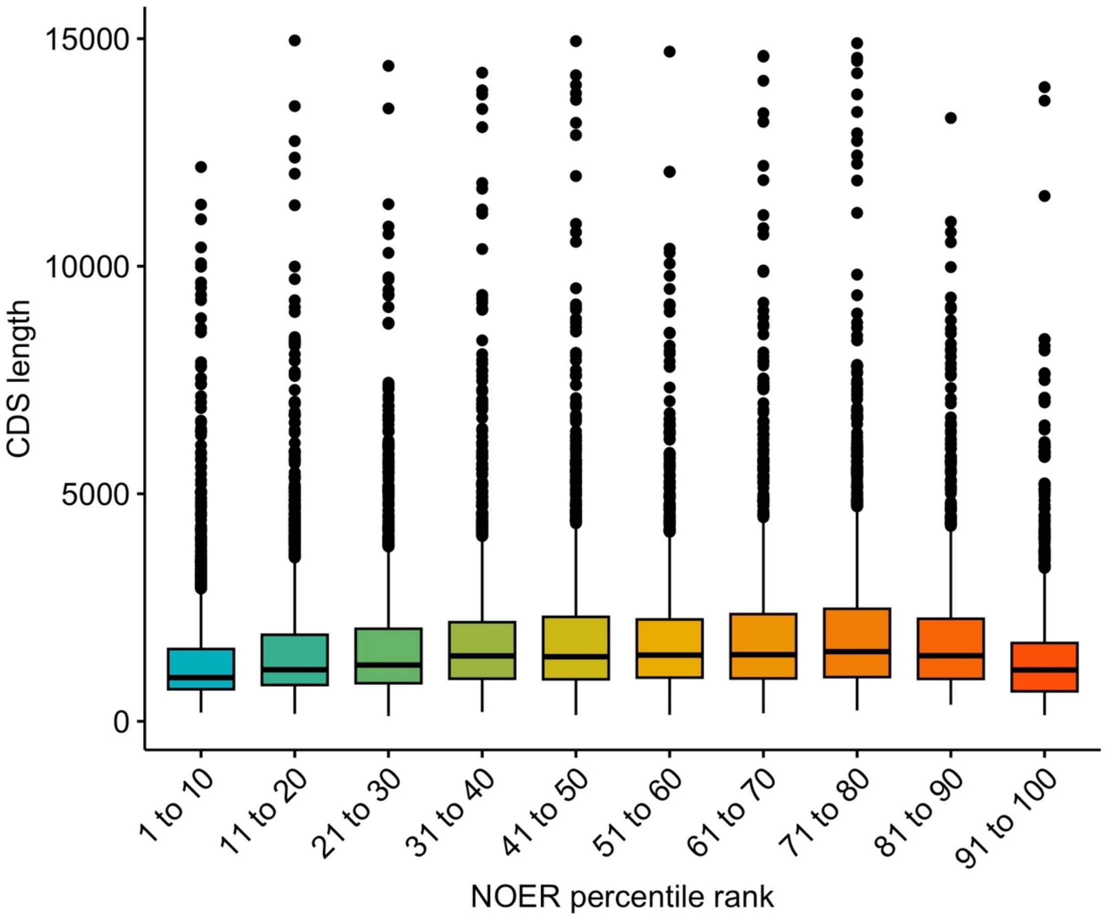

Supplementary Figure 5 – CDS length by NOER percentile rank.

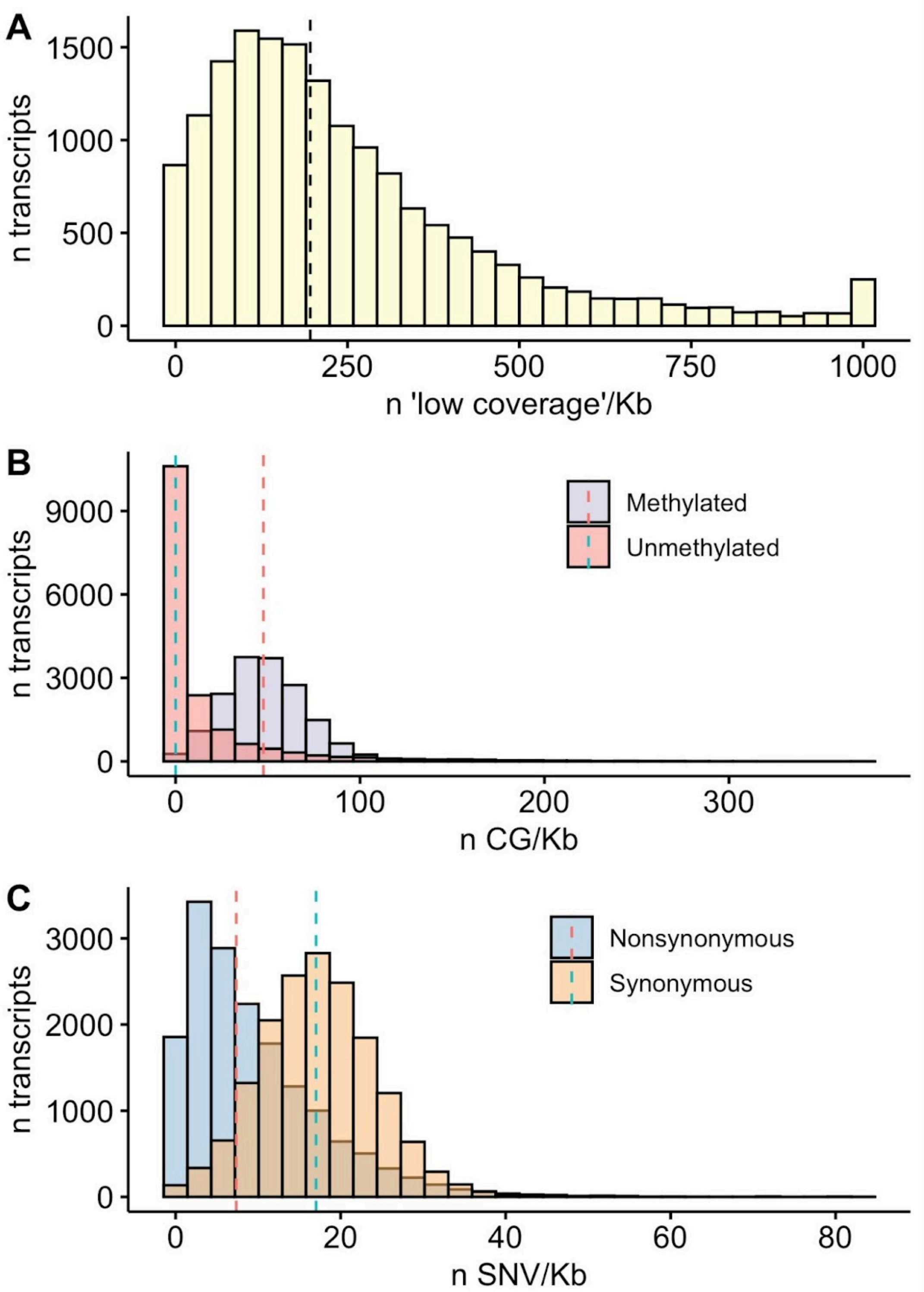

Supplementary Figure 6 – Histograms showing the number of transcripts binned by the number of 'low coverage' positions (A), methylated and unmethylated CG dinucleotides (B), and synonymous and nonsynonymous SNVs (C). Dashed lines represent median values.

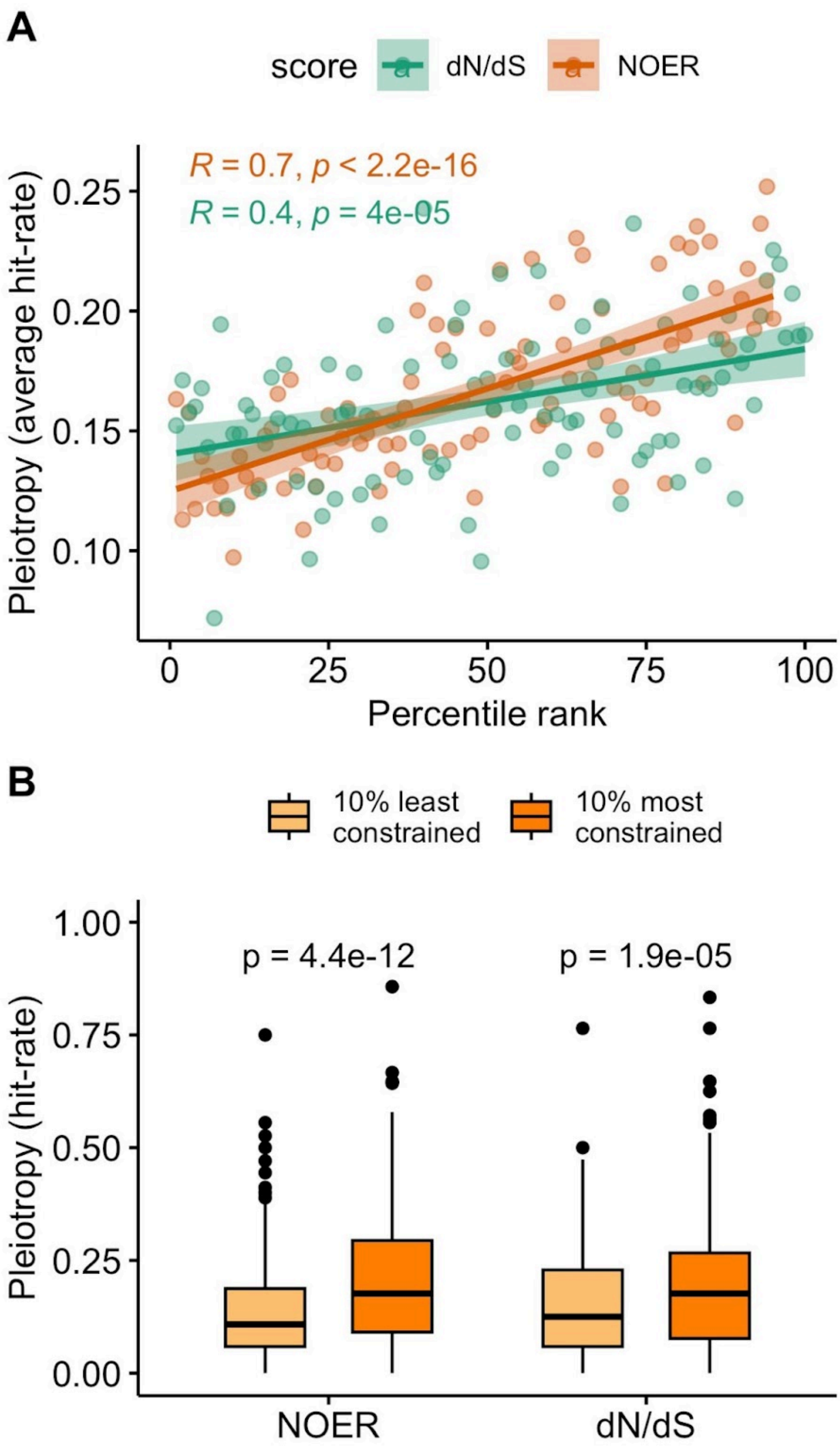

Supplementary Figure 7 – (A) Scatter plot showing the positive Spearman's correlation between constraint and pleiotropy for NOER and dN/dS. Pleiotropy is defined here by hit rate: the number of top-level mouse phenotype ontology terms associated with the knock-out divided by the potential number of top-level terms based on the conducted phenotyping tests. (B) The 10% most constrained mouse genes are significantly more pleiotropic than the 10% least constrained mouse genes for NOER and dN/dS.

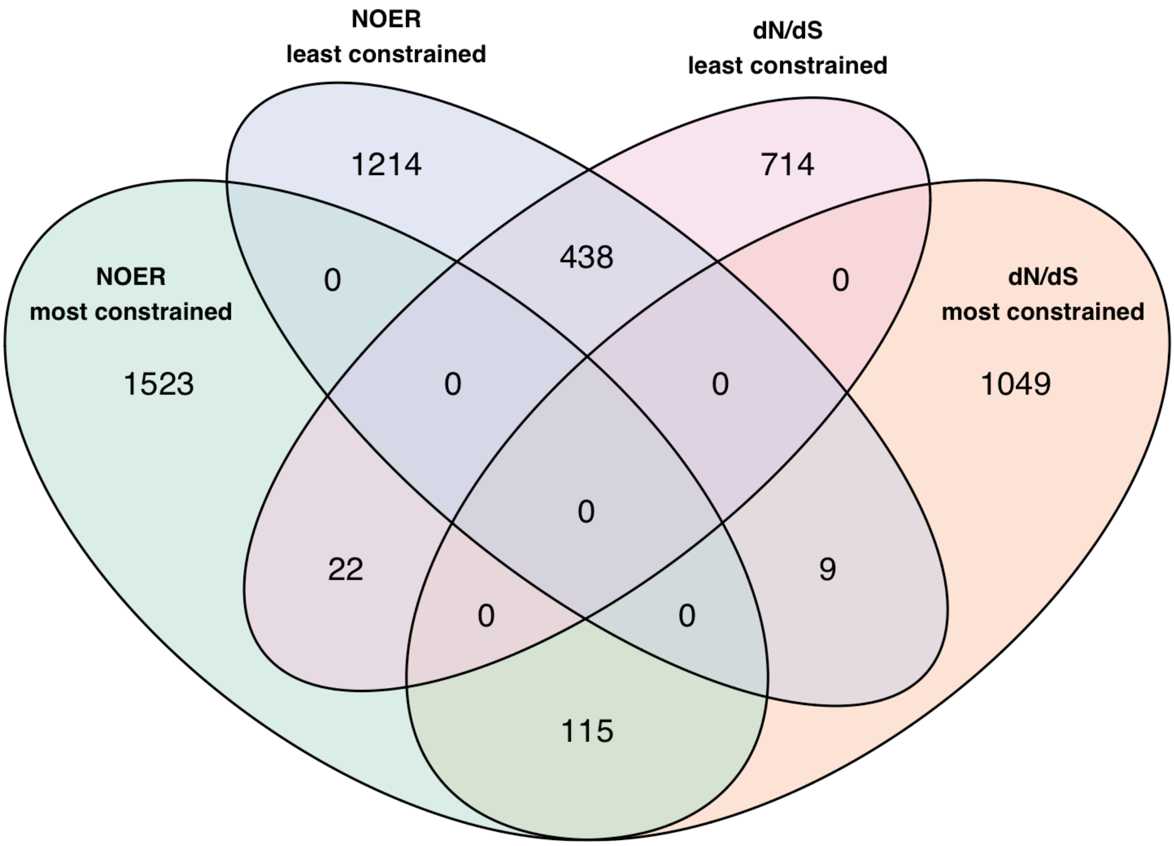

Supplementary Figure 8 – A Venn diagram of the overlap between gene sets including the 10% most constrained genes defined by NOER (n = 1660), the 10% least constrained genes defined by NOER (n = 1661), the 10% most constrained genes defined by *Mus musculus* to *Mus spretus* dN/dS (n = 1174), and the 10% least constrained genes defined by *Mus musculus* to *Mus spretus* dN/dS (n = 1218).

Supplementary Table 1 – A summary of differences between dN/dS, NOER, missense z-score, and LOEUF. Abbreviations: single nucleotide variant (SNV); single nucleotide polymorphism (SNP); allele frequency (AF); protein-truncating variant (PTV); loss-of-function (LoF).

**Score**	**dN/dS**	**Missense z-score**	**Loss-of-functi-on observed/exp-ected upper bound fraction (LOEUF)**	**Nonsynonym-0us observed expected ratio (NOER)**
**Species**	Human and mouse	Human	Human	Mouse
**Brief description**	Gene-specific measure of selection on fixed nonsynonymous variation between species.	Gene-specific measure of tolerance of de novo missense mutations.	Gene-specific continuous measure of selection against pLoF variants.	Gene-specific measure of constraint against nonsynonymous variation.
**Method summary**	Calculated as the ratio of nonsynonymous variants per nonsynonymous site (dN) to synonymous variants per synonymous site (dS).	Based on the difference between the observed and expected number of missense SNVs given no selection. The expected number is predicted using a mutation model trained using synonymous variants.	Based on the ratio between the observed and expected number of LoF variants given no selection. The expected number is predicted using a mutation model was trained using synonymous variants.	Based on the ratio between the observed and expected number of nonsynonymous variants given no selection. The expected number is predicted using a mutation model was trained using synonymous variants.
**Interpretation**	Continuous score >= 0. A lower value indicates more constraint.	Continuous Z-score. A higher value indicates more constraint.	Continuous score >= 0. A lower value indicates more constraint.	Continuous score >= 0. A lower value indicates more constraint.
**Type of variation**	SNPs: synonymous and nonsynonymous.	SNVs: missense and synonymous	PTVs: nonsense, splice, frameshift (predicted LoF with VEP-LOFTEE).	SNVs: synonymous and nonsynonymous.
**Source of variation**	Reference genomes (eg human and chimpanzee, or *Mus musculus* and *Mus spretus*).	6,503 whole exomes from the ESP (human).	125,748 whole exomes from gnomAD (human).	67 whole genomes from 8 populations of wild mice (*Mus musculus sp* and *Mus spretus*).
**AF filter**	NA	Singletons only.	AF < 0.001	NA

**Regional mutability**	Controlled for by the rate of substitutions at synonymous sites (dS).	Controlled for by trinucleotide probabilities of mutation and regional divergence between humans and macaques.	Controlled for by trinucleotide probabilities of mutation. Distinguishes between methylated and unmethylated CpG sites.	Controlled for by trinucleotide probabilities of mutation. Distinguishes between methylated and unmethylated CpG sites.
**Functional correlations**	NA	The most constrained genes are enriched for LoF variants in individuals with autism spectrum disorders or intellectual disability.	The most constrained genes are enriched for haploinsufficient genes; depleted for olfactory genes; enriched for cellular lethal genes; enriched for knockout lethal mouse orthologues; enriched for genes with a higher number of protein-protein interactions; enriched for genes expressed in a greater number of tissues; enriched for LoF variants in individuals with intellectual disability;	The most constrained mouse genes are enriched for genes that result in knockout lethality and sub-viability; pleiotropic genes; and genes with a greater number of pathogenic variant sites in their human orthologues.
**Other notes**	NA	The expected number of missense SNVs were predicted using a linear regression model trained to predict the number of synonymous SNVs as a function of the transcript specific probability of incurring a synonymous substitution (adjusted for	The expected number of pLoF variants is predicted using a mutational model trained on the number of synonymous SNVs. Confidence intervals are calculated for the observed number of variants, with the score based on the upper limit of the 90% interval.	The expected number of nonsynonymous SNVs were predicted using a linear regression model trained to predict the number of synonymous SNVs as a function of the transcript specific probability of incurring a synonymous substitution and number of ‘low coverage’ positions.

		read depth), and the regional divergence between humans and macaques.		
**Reference**	(Herrero et al. 2016; Kryazhimskiy and Plotkin 2008)	(Samocha et al. 2014)	(Karczewski et al. 2020)	

Supplementary Table 2 – Trinucleotide substitution rates.

k3_from	k3_to	k1_from	k1_to	k3_from_N	k3_to_N	k3_mu_rate
AAA	ACA	A	C	36988253	272952	0.00737942394846277
AAA	AGA	A	G	36988253	499206	0.0134963389592907
AAA	ATA	A	T	36988253	210766	0.00569818747589944
AAC	ACC	A	C	20292315	165834	0.00817225634433528
AAC	AGC	A	G	20292315	694575	0.03422847516412
AAC	ATC	A	T	20292315	179830	0.00886197558041061
AAG	ACG	A	C	29050888	280726	0.00966325022491567
AAG	AGG	A	G	29050888	457530	0.0157492604012655
AAG	ATG	A	T	29050888	226203	0.00778644012534144
AAT	ACT	A	C	27701785	232018	0.00837556135823017
AAT	AGT	A	G	27701785	912901	0.0329545911933112
AAT	ATT	A	T	27701785	270585	0.00976778211223573
ACA	AAA	C	A	31283343	461370	0.0147481041268512
ACA	AGA	C	G	31283343	294623	0.00941788734023726
ACA	ATA	C	T	31283343	1993235	0.0637155370511393
ACC	AAC	C	A	16096941	296366	0.0184113242385618
ACC	AGC	C	G	16096941	177005	0.0109961886547264
ACC	ATC	C	T	16096941	971319	0.0603418376199552
ACG	AAG	C	A	3054234	68204	0.0223309674373345
ACG	AGG	C	G	3054234	59228	0.0193920963488718
ACG	ATG	C	T	3054234	1170001	0.383075101645781
ACT	AAT	C	A	23984695	313119	0.013054950250566
ACT	AGT	C	G	23984695	300753	0.0125393714616759
ACT	ATT	C	T	23984695	1269478	0.0529286697204196
CAA	CCA	A	C	24706029	171957	0.00696012297241293
CAA	CGA	A	G	24706029	465911	0.018858190444122
CAA	CTA	A	T	24706029	136398	0.00552083865845053
CAC	CCC	A	C	21789849	179275	0.00822745490342774
CAC	CGC	A	G	21789849	555065	0.0254735588117201
CAC	CTC	A	T	21789849	216532	0.00993728777101668
CAG	CCG	A	C	29610429	201748	0.006813410234617
CAG	CGG	A	G	29610429	734644	0.0248103126097903
CAG	CTG	A	T	29610429	235508	0.00795354906880951
CAT	CCT	A	C	27803613	191380	0.0068832780833196
CAT	CGT	A	G	27803613	1123596	0.040411870212695

CAT	CTT	A	T	27803613	282610	0.0101645063179379
CCA	CAA	C	A	24505623	290220	0.0118429961972401
CCA	CGA	C	G	24505623	126258	0.00515220527141873
CCA	CTA	C	T	24505623	1372535	0.0560089821017813
CCC	CAC	C	A	16779190	226255	0.0134842623511624
CCC	CGC	C	G	16779190	104210	0.00621066928737323
CCC	CTC	C	T	16779190	1012396	0.0603364047966559
CCG	CAG	C	A	2358944	57763	0.0244868042649592
CCG	CGG	C	G	2358944	44938	0.0190500495136807
CCG	CTG	C	T	2358944	853464	0.361799177937246
CCT	CAT	C	A	24696596	279445	0.011315122132621
CCT	CGT	C	G	24696596	159274	0.00644922887348524
CCT	CTT	C	T	24696596	1237309	0.0501003863042502
GAA	GCA	A	C	27289227	124878	0.00457609151039712
GAA	GGA	A	G	27289227	569210	0.0208584142013257
GAA	GTA	A	T	27289227	144659	0.00530095630777669
GAC	GCC	A	C	15421892	93236	0.00604569141062588
GAC	GGC	A	G	15421892	545510	0.0353724432773878
GAC	GTC	A	T	15421892	161266	0.010456953011991
GAG	GCG	A	C	23287317	148975	0.00639725907454259
GAG	GGG	A	G	23287317	390147	0.0167536260188325
GAG	GTG	A	T	23287317	174234	0.00748192675008461
GAT	GCT	A	C	18404738	131143	0.00712550213972076
GAT	GGT	A	G	18404738	569794	0.0309590932508792
GAT	GTT	A	T	18404738	177716	0.00965599184297
GCA	GAA	C	A	20550410	423307	0.0205984698115512
GCA	GGA	C	G	20550410	140321	0.00682813627562662
GCA	GTA	C	T	20550410	1103388	0.0536917754925571
GCC	GAC	C	A	14104098	234308	0.0166127603480917
GCC	GGC	C	G	14104098	125761	0.00891662834447123
GCC	GTC	C	T	14104098	972058	0.0689202528229739
GCG	GAG	C	A	1811506	65634	0.0362317320505701
GCG	GGG	C	G	1811506	29766	0.0164316320233
GCG	GTG	C	T	1811506	650700	0.359203888918944
GCT	GAT	C	A	20665243	354037	0.0171320027545769
GCT	GGT	C	G	20665243	173334	0.00838770683703066
GCT	GTT	C	T	20665243	1176862	0.0569488585253994
TAA	TCA	A	C	24687719	165522	0.00670462913159373
TAA	TGA	A	G	24687719	421323	0.0170660967098661
TAA	TTA	A	T	24687719	232601	0.00942172907914255
TAC	TCC	A	C	16495896	131445	0.0079683455812282
TAC	TGC	A	G	16495896	499484	0.030279288860696
TAC	TTC	A	T	16495896	219126	0.0132836676467892
TAG	TCG	A	C	19245954	137873	0.00716373945401719
TAG	TGG	A	G	19245954	374099	0.0194377997578088
TAG	TTG	A	T	19245954	177362	0.00921554733010377
TAT	TCT	A	C	25031734	189866	0.00758501188930819
TAT	TGT	A	G	25031734	816801	0.0326306199962016
TAT	TTT	A	T	25031734	285337	0.0113990105519658
TCA	TAA	C	A	27860961	327366	0.0117499895283583
TCA	TGA	C	G	27860961	178000	0.00638886792167722
TCA	TTA	C	T	27860961	1317087	0.0472735667660566
TCC	TAC	C	A	21534289	260973	0.0121189513152721

TCC	TGC	C	G	21534289	157156	0.00729794236531329
TCC	TTC	C	T	21534289	1424384	0.0661449282119321
TCG	TAG	C	A	2397415	51435	0.0214543581315709
TCG	TGG	C	G	2397415	48309	0.0201504537178586
TCG	TTG	C	T	2397415	750003	0.312838202814281
TCT	TAT	C	A	32622774	413202	0.0126660596060899
TCT	TGT	C	G	32622774	298732	0.00915716119052292
TCT	TTT	C	T	32622774	1406614	0.0431175472692788

Supplementary Table 3 – CG dinucleotide substitution rates by methylation state.

from	to	from_N	to_N	state	mu_rate
C	A	16075014	494263	methylated	0.0307472827084319
C	G	16075014	313493	methylated	0.0195018803716127
C	T	16075014	5994249	methylated	0.372892303546361
C	A	533814	7681	unmethylated	0.0143889069975684
C	G	533814	5704	unmethylated	0.0106853698104583
C	T	533814	26843	unmethylated	0.0502853053685366

Supplementary Table 4 – Neutral model coefficients. The neutral model is a linear regression predicting the number synonymous SNVs as a function of the genes 1) probability of synonymous substitution P(synonymous) and 2) number of ‘low coverage’ positions (N ‘low coverage’).

Coefficient	Estimate	SE	P
Intercept	-0.6252925	0.1406312	8.79e-06
P(synonymous)	0.8575847	0.0033095	< 2e-16
N ‘low coverage’	-0.0100484	0.0002763	< 2e-16
